# Mucormycosis Causing Splenic Infarction, Gastric Fistula, and Brain Abscess in a Patient With Acute Myeloid Leukemia: A Case Report

**DOI:** 10.1155/crdi/4079965

**Published:** 2024-12-18

**Authors:** Fernando S. da Silveira, Rafael Brito Foureaux Ribeiro, Sandra Lucia Branco Mendes Coutinho, Evelin Soares de Brito, Jacques F. Meis, Marcela Santos Corrêa da Costa, Julival Fagundes Ribeiro, Tazio Vanni

**Affiliations:** ^1^Infectious Diseases Service, Hospital de Base, Federal District, Brasilia, Brazil; ^2^Hematology Service, Hospital de Base, Federal District, Brasilia, Brazil; ^3^Pathological Anatomy Service, Hospital de Base, Federal District, Brasilia, Brazil; ^4^Pharmacy Service, Hospital de Base, Federal District, Brasilia, Brazil; ^5^Institute of Translational Research, Cologne Excellence Cluster on Cellular Stress Responses in Aging-Associated Diseases (CECAD), University of Cologne, Cologne, Germany; ^6^Federal District Health Department, Federal District, Brasilia, Brazil; ^7^Hospital Infection Control Department, Hospital de Base, Federal District, Brasilia, Brazil

**Keywords:** fungal infection, gastric fistula, immunocompromised, leukemia, mucormycosis, splenic infarction

## Abstract

Invasive mucormycosis is an aggressive fungal infection characterized by rapid progression, primarily impacting immunocompromised individuals. Herein, we report a case of splenic infarction in association with gastrointestinal fistula and brain abscess as a rare presentation of mucormycosis biopsy, proven in a 56-year-old patient diagnosed with acute myeloid leukemia. The patient initially sought medical attention with a 3-week history of fever, night sweats, and malaise. Considering the chest computed tomography findings compatible with fungal disease and neutropenia, he underwent broad-spectrum antifungal therapy. Following the occurrence of splenic infarctions and a gastric fistula, the patient underwent a partial gastrectomy and splenectomy. Despite the interventions, the patient did not have a successful outcome and died on the second postoperative day. This case highlights the importance of timely suspicion, immediate antifungal therapy, and surgical intervention to improve the survival prospects of patients with multifaceted manifestations of mucormycosis.

## 1. Introduction

Mucormycosis is an invasive fungal infection caused by fungi of the order *Mucorales* and presents a challenge in clinical practice due to its rapid progression, diverse clinical manifestations, and high mortality, ranging from 40% to 80%, depending on the underlying disease and the site of infection [1]. The disease is commonly associated with immunocompromised states such as hematologic malignancies, organ transplantation, or uncontrolled diabetes mellitus [2]. In this report, we present a rare case involving splenic infarction, gastric fistula, and brain abscess in a patient who was newly diagnosed with acute myeloid leukemia. The postmortem diagnosis revealed mucormycosis as the underlying cause. This discussion encompasses the patient's clinical presentation, examination findings, differential diagnosis, treatment, and clinical progression.

The case report was approved by the Ethics Committee of Instituto de Gestão Estratégica do Distrito Federal (IGESDF) (CAAE 67035923.0.0000.8153).

## 2. Case Presentation

A 56-year-old male presented to medical care with a 3-week history of fever, night sweats, weight loss, and malaise. His medical history revealed tegumentary leishmaniasis successfully treated 1 year ago and aplastic anemia 20 years ago. Concerning exposure and epidemiological factors, he resided in a rural region of Brazil and was engaged in daily activities centered around gardening and the care of animals. On physical examination, he had multiple painful oral ulcers and a diffuse decrease in breath sounds. He did not present tenderness, hepatomegaly, or splenomegaly.

Initial laboratory investigations revealed a high white blood cell count of 202,000/*μ*L (with 10,900 blasts), low red blood cell count of 6.6 g/d, and low platelet count of 44,000/*μ*L. Bone marrow immunophenotyping led to the diagnosis of acute myeloid leukemia (M5). Initial chest computed tomography (CT) showed multiple nodules surrounded by a ground-glass attenuation halo. Abdominal CT showed no abnormalities.

The clinical manifestations observed in a patient with oncohematological conditions, in conjunction with findings from chest imaging studies, strongly suggest the presence of fungal infections such as aspergillosis, mucormycosis, and fusariosis. In light of the patient's medical history and epidemiological factors, potential differential diagnoses also encompassed blastomycosis, cryptococcosis, actinomycosis, tuberculosis, nontuberculous mycobacteria, and nocardiosis. A serum test for the galactomannan antigen was conducted, resulting in 0.33 (ELISA). The indirect immunofluorescence test for leishmaniasis (IgM/IgG) yielded negative results. In addition, the bone marrow biopsy did not indicate the presence of leishmania amastigotes. Both fungal and bacterial cultures from the blood tested negative.

Upon observing findings consistent with fungal pneumonia on the chest CT scan in conjunction with febrile neutropenia, the prompt administration of voriconazole 6 mg/kg IV q12h (with a loading dose of two doses), followed by 4 mg/kg IV q12h was initiated on 22 April 2021. In addition, piperacillin-–tazobactam 4.5 gm IV q6h was concomitantly initiated for suspected concurrent bacterial superinfection. In addition, on 26 April 2021, the patient underwent chemotherapy using the daunorubicin + cytarabine (7 + 3) protocol for the treatment of leukemia to induce remission.

**Figure 1:**
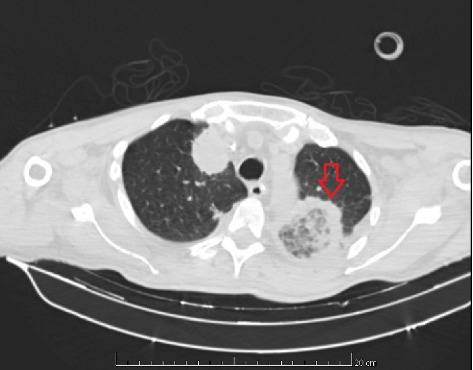
Chest CT scan showing an inverted halo sign on the left upper lobe (red arrow) characterized by a central ground-glass opacity surrounded by a peripheral rim of consolidation. This radiologic finding is often associated with a range of pulmonary conditions, including angioinvasive fungal infections (e.g., invasive aspergillosis and mucormycosis), organizing pneumonia, and pulmonary infarction.

On 14 May 2021, due to the deterioration of the pulmonary condition in the presence of neutropenia, despite treatment with voriconazole, and the presence of a black skin lesion on the inner face of the left lower limb consistent with fusariosis, the antifungal was switched to liposomal amphotericin B at a dosage of 5 mg/kg IV daily. A skin biopsy was also conducted at this time. In this context, the patient required admission to the intensive care unit (ICU) for ventilatory support. Following 14 days of liposomal amphotericin B treatment and worsening ventilatory parameters, a repeat chest CT revealed a rounded area of ground-glass opacity surrounded by a ring of consolidation on the left upper lobe, consistent with the reversed halo sign, as evidenced in Figure 1.

**Figure 2:**
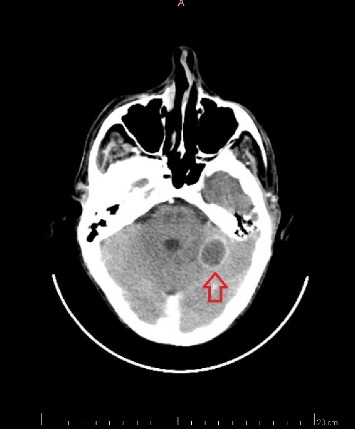
Axial CT scan of the brain showing a hypodense, rounded lesion in the left frontal gyrus (red arrow), consistent with a cerebral abscess. The lesion exhibits a central low-density area, indicative of necrotic or liquefied content, surrounded by a thin rim, which typically represents the capsule of the abscess.

The CT of the abdomen on the same day showed a spleen with diffusely reduced and heterogeneous density, which could be compatible with collection or infarction. A brain CT was also performed and showed a nodular lesion with peripheral contrast enhancement in the left frontal gyrus and cerebellar hemisphere associated with vasogenic edema suggestive of the fungal abscess, as demonstrated in Figure 2. A bronchoscopy with bronchoalveolar lavage was requested but not performed due to thrombocytopenia and risk of bleeding.

On 08 June 2021, the patient underwent chest drain placement. Due to clinical deterioration and changes in chest drain secretion, a methylene blue test was conducted, revealing a fistula connecting the gastric fundus/cardia to the base of the left hemithorax. Subsequently, the patient underwent exploratory laparotomy, leading to partial gastrectomy, and gastropleural and splenic biopsy. Despite treatment with liposomal amphotericin B, surgical intervention, and supportive care, the patient passed away on the second postoperative day.

**Figure 3:**
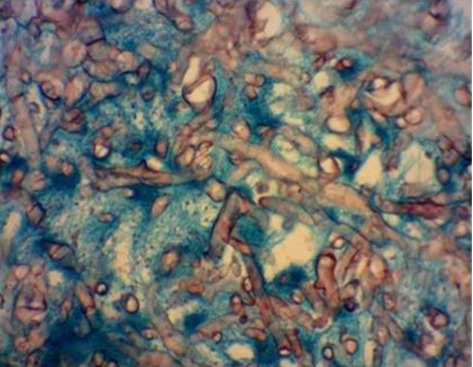
Histopathological section showing wide, nonseptate fungal hyphae with variable diameters and characteristic 90° angle branching, indicative of angioinvasive fungal infection consistent with *Mucorales* species. Stained with Grocott's methenamine silver (GMS) stain, which highlights the fungal structures. Image captured at 400x magnification (40x objective lens and 10x eyepiece).

**Figure 4:**
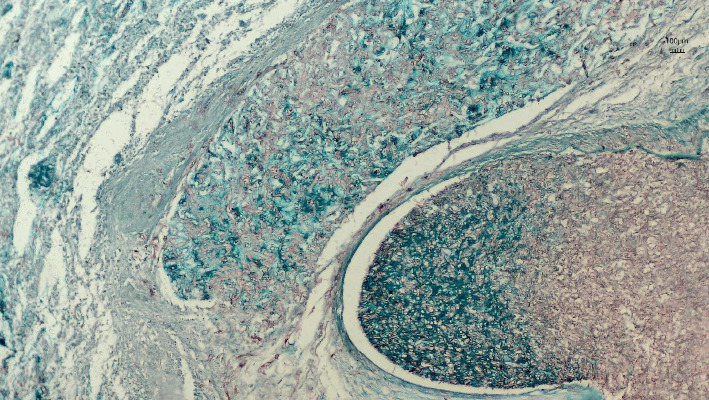
Cross-section of an artery showing hyphae infiltrating the vessel wall. The fungal hyphae are visible within the arterial structure, displaying irregular branching patterns consistent with angioinvasive fungal infection. Stained with Grocott's methenamine silver (GMS), which highlights the fungal elements. The image was captured at 200x magnification (20x objective lens and 10x eyepiece), with a 100 *μ*m scale bar visible in the upper right corner.

Histopathological examination of the surgical excision during the postmortem period identified mucormycosis in the gastric fistula and septic infarction in the spleen due to angioinvasive mucormycosis, as evidenced in Figures 3 and 4. The skin biopsy results were negative for fungal structures (PAS stain).

## 3. Discussion

Neoplasia patients exhibit an increased susceptibility to fungal infections due to both disease and treatment immunosuppression. Many neoplasms, along with the common comorbidities observed in oncologic populations, lead to immune dysregulation either directly or indirectly [3]. Factors such as neutropenia, mucosal barrier disruption from chemotherapy, and the administration of corticosteroids and other immunosuppressive agents create an environment conducive to the proliferation of fungal pathogens, including *Aspergillus* spp., *Candida* spp., and *Mucorales* [4]. While both solid tumors and hematologic malignancies elevate the risk for fungal infections, hematologic malignancies often present with greater susceptibility due to often prolonged neutropenia, a significant predisposing factor for fungal infections, as neutrophils play a crucial role in the defense against invasive fungi [5].

Fungal infections, particularly those caused by invasive molds such as *Mucorales*, represent a critical challenge in immunocompromised populations due to their rapid progression and high mortality rates. Mucormycosis, a rare but increasingly recognized infection, has shown rising incidence, especially among patients with hematologic malignancies, diabetes mellitus, or those undergoing immunosuppressive therapy [6]. The angioinvasive nature of *Mucorales* fungi leads to extensive tissue necrosis and hematogenous dissemination, which contributes to the often fatal outcomes [4]. Despite advances in antifungal therapy, the mortality of mucormycosis remains high, with rates between 40%–80%, contingent on host factors and infection site [1]. Prompt recognition and aggressive management are therefore essential to improve patient survival in cases where early diagnosis is challenging.

The case report provides an account of mucormycosis in a 56-year-old patient with acute myeloid leukemia who presented with gastric fistula, splenic infarction, and brain abscess. The causal agent was not initially identified through laboratory and microbiological tests. Etiological confirmation was only possible postmortem through anatomopathological examination of gastric and splenic biopsy specimens. The diagnosis of brain abscess due to mucormycosis was considered presumptive, as a biopsy of the brain lesion was not performed. This determination was made based on the concordance of the brain lesion visualized in the brain CT with a fungal abscess, combined with identifying *Mucorales* structures in extracerebral tissues.

Mucormycosis caused by various *Mucorales* genera or species presents similar clinical patterns. *Rhizopus* spp., *Lichtheimia* spp., *Rhizomucor* spp., and *Mucor* spp. are the most commonly isolated organisms in mucormycosis cases [3]. The involvement of multiple sites in this case underscores the invasive nature of mucormycosis, particularly in the presence of immunosuppression and neutropenia resulting from hematological malignancy. In such instances, the ability to control the fungus's expansion is compromised, emphasizing the aggressive potential of the condition [7].

It is important to highlight that galactomannan detection is not evident in mucormycosis infection unlike in aspergillosis [8]. In patients with hematological malignancy and pulmonary infection, the presence of a reversed halo sign in the pulmonary CT scan, characterized by an area of ground-glass opacity surrounded by a ring of consolidation, strongly indicates mucormycosis, prompting the initiation of appropriate therapy [9]. It is essential to note that mucormycosis does not respond to voriconazole, a commonly initiated antifungal drug in suspected fungal pulmonary infections in neutropenic patients [10]. High-dose liposomal amphotericin B is the recommended first-line treatment, with isavuconazole and posaconazole serving as alternative salvage treatment options [10].

Gastrointestinal manifestations of mucormycosis represent only 7% of all cases, with the stomach being the most frequently affected organ. Splenic involvement is much rarer, with limited reported cases in the literature [1, 11, 12].

In the presence of nonspecific findings in immunocompromised hematological patients, clinicians should consider the possibility of splenic mucormycosis, particularly when accompanied by splenic lesions suggestive of abscess or infarction on imaging results. In addition, the diagnosis of gastric fistula should be entertained in patients presenting with symptoms such as melena, hematemesis, abdominal distention, or abdominal pain. In the context of an immunocompromised host, gastric mucormycosis should be included in the differential diagnosis of refractory gastric ulcers. Obtaining a gastric biopsy and exudate culture is recommended to confirm *Mucorales* infection. Moreover, radiological findings indicative of pleural effusion, especially with volume increase despite therapy, may raise suspicion of a gastropleural fistula. In addition, within this context, it is advisable to broaden the investigation of other foci of fungal infection, including the central nervous system, due to the potential for cerebral abscess through hematogenous dissemination.

## 4. Conclusion

We report a case of disseminated mucormycosis complicated by gastropleural fistula, splenic infarction, and brain abscess. While each manifestation is individually rare, their collective occurrence should prompt consideration of an underlying opportunistic infection. The management of these cases should encompass a multidisciplinary approach, incorporating antifungal therapy to address the mucormycosis infection, supportive care to manage complications, and surgical interventions to address the gastric fistula and associated issues.

## Data Availability

The data that support the findings of this study are available on request from the corresponding author. The data are not publicly available due to privacy or ethical restrictions.
